# Association between the triglyceride-glucose index and kidney stone disease: a systematic review and dose-response meta-analysis

**DOI:** 10.1186/s12944-026-02994-3

**Published:** 2026-06-12

**Authors:** Shiva Maleki, Bahar Darouei, Alireza Amin, Ali Amani-Beni, Arsham Seifnezhad, David J Tunnicliffe, Javier A. Neyra, Reza Amani-Beni

**Affiliations:** 1 Isfahan Kidney Diseases Research Center, Isfahan University of Medical Sciences, Isfahan, Iran; 2 Isfahan Cardiovascular Research Center, Cardiovascular Research Institute, Isfahan University of Medical Sciences, Hezar Jerib Avenue, Isfahan, Isfahan Province Iran; 3 Water and Electrolytes Research Center, Isfahan University of Medical Sciences, Isfahan, Iran; 4 Urology and Nephrology Research Center, Research Institute for Urology and Nephrology, Center of Excellence in Urology, Shahid Labbafinejad Medical Center, Shahid Beheshti University of Medical Sciences, Tehran, Iran; 5 Sydney School of Public Health, The University of Sydney, Sydney, NSW Australia; 6 Centre for Kidney Research, The Children’s Hospital at Westmead, Westmead, NSW Australia; 7 Department of Medicine, Division of Nephrology, University of Alabama at Birmingham, Birmingham, AL USA

**Keywords:** Triglyceride-glucose index, Insulin resistance, Nephrolithiasis, Triglycerides, Blood Glucose, Meta-Analysis as Topic

## Abstract

**Background:**

The triglyceride-glucose (TyG) index is an accessible marker of insulin resistance and may reflect metabolic susceptibility to kidney stone disease (KSD). This review synthesizes observational evidence on TyG and KSD and examines whether the association varies with increasing TyG.

**Methods:**

PubMed, Embase, Web of Science, Scopus, the Cochrane Library, and ProQuest were searched up to May 22, 2026 according to PRISMA 2020 and a PROSPERO-registered protocol. Eligible studies enrolled adults, assessed TyG as either a continuous or categorical exposure, reported KSD as the outcome, and provided extractable effect estimates. Study quality was evaluated using the Joanna Briggs Institute (JBI) tools. Maximally adjusted odds ratios (ORs) were synthesized using the restricted maximum likelihood (REML) random-effects model. One-stage dose-response meta-analyses using linear and restricted cubic spline models were also conducted, and the certainty of evidence was judged using GRADE.

**Results:**

Fourteen studies (total *n* = 1,066,215; KSD cases = 50,286) met the inclusion criteria. In the continuous analysis, each 1-unit increment in TyG corresponded to higher KSD odds (pooled adjusted OR: 1.33; 95% confidence interval [CI]: 1.15–1.54). Compared with the lowest TyG category, the highest had 52% higher odds of KSD (OR: 1.52; 95% CI: 1.26–1.84). In a one-stage random-effects dose-response meta-analysis, restricted cubic spline models provided the best fit but showed no statistical evidence of non-linearity, supporting a linear association with a 26% increase in odds per 1-unit increase in TyG (OR: 1.26; 95% CI: 1.12–1.42; 95% PI: 0.91–1.75).

**Conclusion:**

A higher TyG index was associated with greater odds of KSD in a broadly linear manner; however, the marked heterogeneity across studies suggests that TyG should be viewed as a potential metabolic risk marker rather than a routine screening tool. These findings may support broader cardiometabolic risk assessment and standard preventive counseling in patients at risk of KSD.

**Supplementary Information:**

The online version contains supplementary material available at 10.1186/s12944-026-02994-3.

## Introduction

Kidney stone disease (KSD), also referred to as nephrolithiasis, develops when urinary constituents exceed their solubility threshold and form crystals that may enlarge or aggregate within the renal collecting system [1]. Its public health importance has increased over the recent decades because both its occurrence and recurrence remain substantial in many populations [2, 3]. Current estimates suggest that approximately 10–15% of adults experience KSD during their lifetime [2–4], and recurrence is frequent, with nearly half of the affected individuals developing another stone episode within five years [5].

The clinical impact of KSD extends beyond acute symptoms, such as renal colic and hematuria. Kidney stones may be complicated by urinary tract infection, pyelonephritis, obstruction, hydronephrosis, and, in some patients, long-term deterioration in kidney function [6–10]. Increasing evidence also supports the concept that KSD is not only a localized urological condition but also part of a broader metabolic phenotype. Obesity, metabolic syndrome, diabetes, insulin resistance (IR), and dyslipidemia have all been linked to the risk [11–15]. IR may contribute to lithogenesis through inflammatory and metabolic pathways that alter the urinary chemistry. Proposed mechanisms include reduced urinary pH, changes in lithogenic solute excretion, and possibly increased urinary calcium excretion in certain settings [16–19]. Previous meta-analyses have further indicated that metabolic syndrome and its components, including higher fasting plasma glucose (FPG), elevated body mass index (BMI), and dyslipidemia, are associated with nephrolithiasis, with the risk appearing to increase as metabolic abnormalities accumulate [20, 21].

The triglyceride-glucose (TyG) index, calculated from fasting triglyceride (TG) and FPG levels, is widely used as an accessible surrogate marker of IR [22, 23]. Higher TyG values have been associated with several cardiometabolic and renal outcomes, including diabetes mellitus, cardiovascular disease, and chronic kidney disease [24–26]. More recently, observational studies have examined whether TyG is related to KSD, but the available findings are not fully consistent. Several studies have reported a higher prevalence of stones in individuals with elevated TyG [13, 27–29]. However, one population-based study found this association mainly in men [30], whereas a National Health and Nutrition Examination Survey (NHANES) analysis restricted to adults without diabetes did not identify an independent association after multivariable adjustment [31].

This inconsistency leaves an important evidence gap: whether TyG represents a reproducible metabolic signal for KSD across populations and whether any association changes progressively across the TyG distribution. The hypothesis of the present review was that higher TyG is associated with greater odds of KSD, reflecting the contribution of IR-related metabolic dysfunction to stone formation. The novel aspect of this study is the integration of available observational evidence with formal dose-response modelling, allowing both the strength and potential shape of the TyG index-KSD association to be evaluated. Therefore, this systematic review and dose-response meta-analysis was designed to synthesize the current evidence on TyG and KSD, quantify the association across continuous and categorical exposure definitions, and explore whether a dose-dependent pattern is present.

## Materials and methods

### Protocol and registration

The review was planned and reported according to the PRISMA 2020 statement [32]. A prespecified protocol describing the review question, eligibility framework, and analytic approach was available in PROSPERO under the registration number CRD420251176773.

### Eligibility criteria

Studies were eligible if they used an observational design, including cohort, case-control, or cross-sectional designs, enrolled adults aged ≥ 18 years, and assessed the TyG index in relation to KSD. No restrictions were applied to the country, setting, or publication language. Eligible studies could model TyG either as a continuous exposure or as study-defined categories; for categorical analyses, the lowest TyG group was used as the reference. Outcomes included prevalent or incident KSD, determined using imaging, clinician diagnosis, procedure/registry/ICD codes, or documented stone passage. In cohort studies, TyG must be measured before the incident KSD during follow-up. Studies were required to report the estimated effect of the TyG index on KSD.

We excluded interventional, ecological, modelling, and review articles; studies restricted to children or adolescents under 18 years of age or to pregnant or gestational diabetes populations; reports without a kidney stone outcome in the renal or upper urinary tract or limited to bladder stones only; studies without TyG measurement or without effect estimates; animal, in vitro, and genetic association studies; and duplicate or overlapping publications, with the report including the largest population retained. For studies based on the NHANES, survey cycles, recruitment years, and age ranges were cross-checked, and it was confirmed that there was no overlap between the included analyses. For NHANES-based publications, survey cycles, recruitment periods, and age ranges were checked to prevent the duplicate inclusion of the same participants. If eligible reports used identical or partially shared NHANES cycles, one estimate was chosen for each synthesis, prioritizing the largest eligible population, most fully adjusted model, and most informative analysis. This principle was also applied to other datasets with potential sample overlap; independent strata were extracted where possible; otherwise, a single best-supported estimate was retained to preserve statistical independence.

Studies that calculated TyG using non-standard formulas or scales were excluded. The standard formula used was TyG = ln [(fasting TG (mg/dL) × FPG (mg/dL)) / 2], which allowed the harmonization of continuous estimates and comparability of categorical contrasts [24]. Non-English and gray literature reports were eligible if they met the abovementioned criteria and provided extractable data for analysis.

### Information sources and search strategy

A systematic literature search was performed in PubMed, Embase, Web of Science, Scopus, ProQuest, and Cochrane Library. The Cochrane Database of Systematic Reviews was used only for the citation tracking. The initial search was conducted on July 21, 2025, and updated on May 22, 2026. No restrictions were imposed on the publication year or language. Gray literature was also considered eligible, including dissertations, theses, conference abstracts or proceedings, preprints such as medRxiv and bioRxiv, and other relevant non-indexed reports. The search syntax combined terms for KSD with terms for TyG and was adapted for each database using both controlled vocabulary and free-text terms, including Medical Subject Headings in MEDLINE and EMTREE terms in Embase (Table S1). Additional records were sought by searching Google Scholar and Google, reviewing the reference lists of the included articles and related reviews, and contacting the corresponding authors when the required information was unavailable.

### Study selection

Retrieved records were managed using EndNote, and duplicate citations were removed using automated detection followed by manual verification. Titles, abstracts, and subsequently, full texts were assessed independently by two reviewers (S.M. and A.A.) against the predefined eligibility criteria. Discrepancies were resolved through discussion, and a third reviewer (R.A.B.) was consulted when necessary.

### Data extraction

Using a piloted Excel sheet, two reviewers (S.M. and B.D.) independently extracted the following study identifiers (country or region, year), design and data source (hospital health examination center, national survey, biobank, occupational), population and enrollment period, total sample size, number of stone events, and stone ascertainment method. Baseline characteristics were recorded as follows: mean age, percentage of men, mean BMI, diabetes prevalence, and hypertension prevalence. Exposure metrics included TyG modeled continuously (per 1-unit or per 1-standard deviation (SD), as available) and category definitions with cut-points or medians and group counts to enable dose-response analyses. For each analysis, the effect measure (risk ratio [RR], hazard ratio [HR], or odds ratio [OR]) with 95% confidence intervals (CIs), model specifications (crude and maximally adjusted), and all covariates used for adjustment were recorded. Discrepancies were resolved by consensus with the third author (R.A.B.).

### Risk of bias assessment

Two reviewers (S.M. and A.S.) independently assessed the risk of bias of each included study using the Joanna Briggs Institute (JBI) design-specific checklists for analytical cross-sectional, case-control, and cohort studies [33]. Item-level judgments were recorded as yes, no, unclear, or not applicable. The assessment addressed potential bias in observational association studies, rather than prognostic accuracy. Any disagreement was resolved by consensus, with the involvement of a third reviewer (R.A.B.) when needed.

For scoring purposes, each “yes” response was assigned 1 point, whereas “no” and “unclear” responses were assigned zero points. Study-level risk of bias was categorized as low risk when at least 70% of items were rated “yes,” moderate risk when 50–69% were rated “yes,” and high risk when 49% or fewer were rated “yes” [34]. Based on the number of items in each checklist, these thresholds corresponded to scores of 6–8 for low risk, 4–5 for moderate risk, and 0–3 for high risk, respectively, in cross-sectional studies; 7–10, 5–6, and 0–4, respectively, in case-control studies; and 8–11, 6–7, and 0–5, respectively, in cohort studies.

### Statistical analysis

A meta-analysis of the outcomes was conducted using random-effects models and restricted maximum likelihood (REML). The effects were synthesized on the log-OR scale. Only one included study reported an HR [35]; because the KSD incidence in that cohort was ~ 1.1%, the HR was treated as approximately equivalent to an OR under the rare-outcome assumption and the estimate was included on the log-OR scale. Pooled estimates with 95% CIs, between-study heterogeneity (I²), and 95% prediction intervals were reported. For continuous exposure, the change in KSD odds was modeled per 1-unit increase in TyG, rescaling estimates reported per SD to a 1-unit increment when necessary. For categorical exposure, the contrast between the highest and lowest TyG categories was pooled. When a study reported several TyG-related estimates (e.g., sex-specific results or separate multiple categorizations), the most fully adjusted overall estimate was prioritized for analysis. This was done only when the strata were mutually non-overlapping; therefore, each participant contributed to only one stratum-specific estimate. If only stratum-specific estimates were reported, they were combined using a fixed-effects meta-analysis to obtain a single study-level estimate for inclusion in the main meta-analysis.

The primary analyses were based on maximally adjusted estimates, whereas crude estimates were synthesized separately as secondary analyses. Heterogeneity was explored using prespecified subgroup analyses according to the geographic region of the study population or data source, diagnostic method, and study size. The geographic region was categorized as East Asia versus non-East Asia, the diagnostic method as imaging-based versus self-report or International Classification of Diseases codes, and the study size as ≥ 40,000 versus < 25,000 total participants. For each subgroup analysis, subgroup-specific within-subgroup heterogeneity, the Q statistic for subgroup differences, and the derived between-group I² were reported. The between-group I² was calculated as max (0, [(Qb − df)/Qb]) × 100. Because all included studies were rated as having a low risk of bias using the Joanna Briggs Institute design-specific checklists, stratification by risk-of-bias category was not performed.

Random-effects meta-regression was performed when at least five studies were available, using the mean age, male proportion, and hypertension prevalence as moderators. Sensitivity analyses included restriction to cross-sectional studies, leave-one-out influence analyses, repeated pooling limited to studies reporting TyG effects per 1-unit increase, exclusion of estimates converted from per 1-SD, and exclusion of preprints or non-peer-reviewed reports. Additional sensitivity analyses were restricted to studies whose maximally adjusted models included core covariates, including age, sex, and BMI. Further restricted analyses were performed after adjustment for kidney function markers, glycemic measures, uric acid or hyperuricemia, lipid profile, or TyG components, including FPG and TG, to assess robustness to differences in adjustment sets and potential over-adjustment. Small-study effects (publication bias) were evaluated using funnel plots and Egger’s regression, with Begg’s test and trim-and-fill used as supportive analyses.

For the dose-response analysis, a one-stage random-effects meta-regression was used to estimate both linear and non-linear associations between TyG and KSD. Non-linearity was evaluated using quadratic, cubic, and restricted cubic spline (RCS) dose-response models. The best-fitting model was selected using Akaike’s information criterion (AIC). RCS models used three knots placed at the 10th, 50th, and 90th percentiles of the TyG distribution, and non-linearity was tested using a Wald test, with *P* < 0.05 considered evidence of a non-linear association [36, 37]. For the linear one-stage random-effects dose-response model, a 95% prediction interval was calculated on the log-OR scale as β ± 1.96√(SE(β)² + τ²), and the limits were exponentiated. Sensitivity analyses were performed to repeat the dose-response assessment using a two-stage approach and crude estimates. For interpretability, the plotting reference value was based on the mean of the study-level representative TyG values in the lowest exposure category among studies contributing to the dose-response meta-analysis (mean ≈ 8; range = 7.83 to 8.53), corresponding approximately to a TG of 70 mg/dL and FPG of 85 mg/dL using the standard TyG formula. All analyses were performed using Stata 17 (StataCorp LLC, College Station, TX, USA) with a two-sided α = 0.05.

### Certainty of evidence

GRADE was applied to nonrandomized association evidence, starting from low certainty. The risk of bias, inconsistency, indirectness, imprecision, and small-study effects were evaluated. Potential upgrades were assessed for dose-response evidence, large magnitude of effect, and all residual confounding would reduce the observed effect [38, 39]. Summary of findings tables were prepared for KSD outcomes.

## Results

### Search results and study selection

**Fig. 1:**
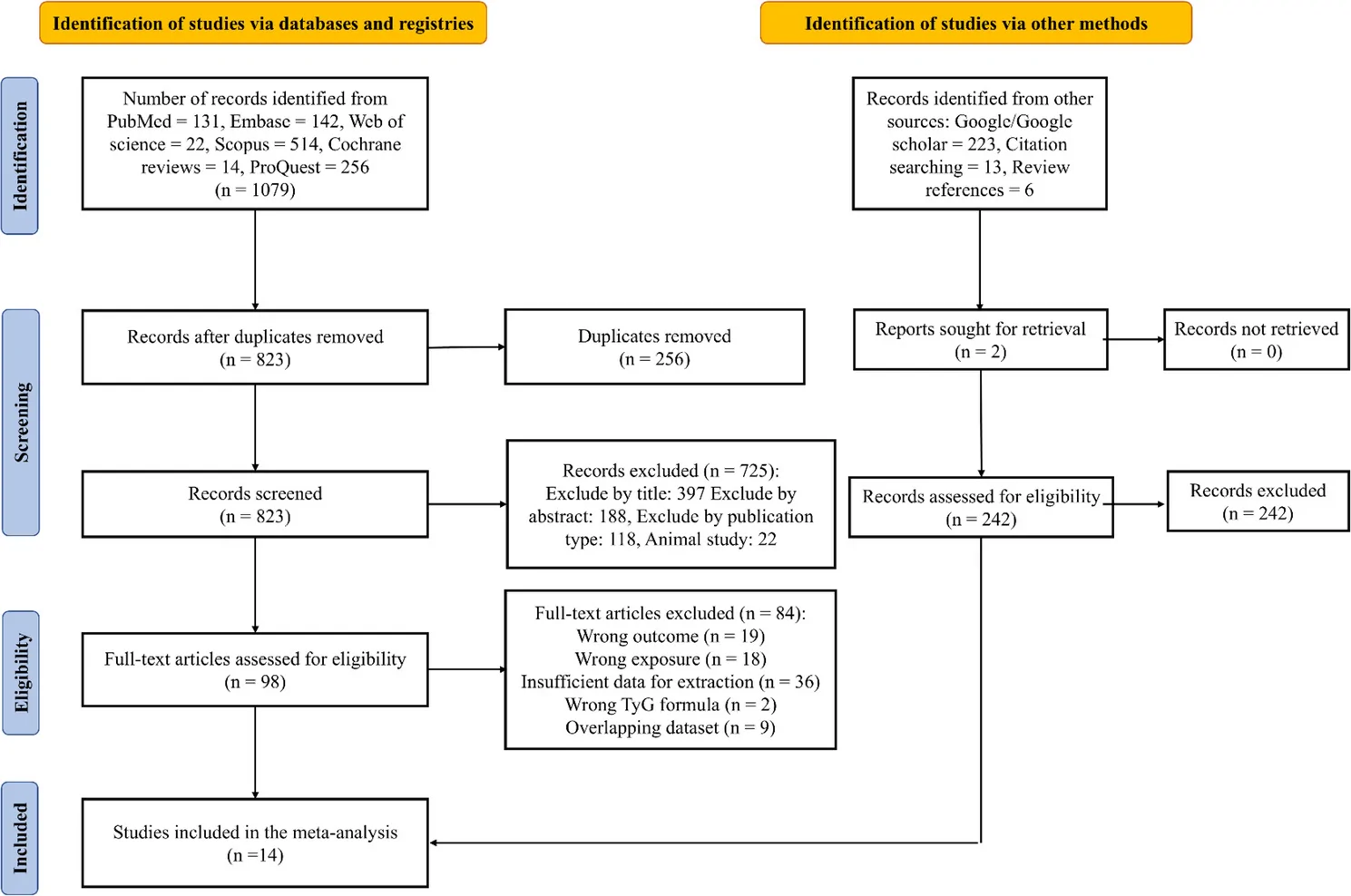
PRISMA flowchart of the included studies

We identified 1079 records from electronic databases. An additional 242 records were identified from other sources (Google Scholar, citation tracking, and reference lists). After removing 256 duplicate records from database searches, 823 unique database records were screened by title or abstract, of which 725 were excluded. We then assessed 98 database records in full text. In total, 14 studies fulfilled all criteria and were included in the qualitative synthesis and meta-analysis [13, 27, 28, 30, 31, 35, 40–47]. A PRISMA flow diagram summarizing the inclusion process is provided in Fig. 1.

### Characteristics of the included studies in the meta-analysis

Fourteen observational studies were included: 12 cross-sectional studies, one case-control study, and one prospective cohort study. These studies were conducted in China (*n* = 9), Taiwan (*n* = 1), and the U.S. NHANES datasets (three enrollment periods), and the UK Biobank [13, 27, 28, 30, 31, 35, 40–47] (Table 1). Across all studies, the combined sample comprised 1,066,215 adults, among whom 50,286 had KSD (crude prevalence = 4.7%). The sample sizes ranged from 414 to 413,209, and the mean age ranged from 40.7 to 57.9 years. Men accounted for 35.9% to 90.6% of participants. Cardiometabolic profiles, including diabetes and hypertension prevalence, varied between studies, as shown in Table 1. The mean BMI ranged from 23.8 to 28.8 kg/m². Kidney stone ascertainment was predominantly ultrasound-based in health examination settings (seven studies, including Doppler variants), with four self-report datasets (three NHANES waves and Taiwan Biobank), one registry/ICD-coded cohort (UK Biobank), and two studies using multimodality imaging or surgical confirmation (Table 1). Fasting status reporting was inconsistent across the included studies. Only a minority explicitly documented fasting procedures, whereas several studies did not clearly report how the fasting status was verified (Table S2). All included studies were appraised as having a low risk of bias using the JBI design-specific checklists. Domain-level judgments are provided in Tables S3-S5. Across studies, adjusted models typically included age and sex, with additional adjustment variables including BMI, hypertension, diabetes, lipid measures, and kidney-related factors. The full adjustment sets are provided in Table S6.

**Table 1:** Baseline characteristics of the included studies

First Author	Study design	Population	Diagnosis method	Total sample size/KSD events	Mean age	Male, %	DM, %	HTN, %	Mean BMI	Analysis	ROB
Liu et al., 2026 [47]	Cross-sectional	Department of Health Management & Institute of Health Management, Sichuan Provincial People’s Hospital from 2015–2021	Color doppler ultrasound	87,399/2575	45.28	53.6%	0%	6.28%	23.8	Categorical	Low
Wang et al., 2024 [13]	Case-control	Chinese adults underwent physical examinations in Ruijin Hospital, Pudong New Area People’s Hospital, and Shanghai Civil Aviation Hospital from 2020–2022	Renal ultrasonography performed by healthcare professionals; stones identified by high echogenicity with posterior acoustic shadowing	117,757/11,645	55.24	51.8%	6.7%	26.5%	24.3	Categorical Continuous DRMA	Low
Zheng et al., 2024 [44]	Cross-sectional	Medical records of adults from the Yangzhou University in 2022	Renal ultrasonography performed by experienced sonographers; stones identified as strong echogenic foci with posterior acoustic shadowing	84,968/5618	46.61	56.2%	NA	32.4%	24.1	Categorical Continuous DRMA	Low
Xiong et al., 2024 [43]	Cross-sectional	Subjects based on the annual health check-up data of employees from a construction company in Hubei in 2022	Color Doppler ultrasound	2490/854	40.71	90.6%	NA	NA	25.7	Categorical DRMA	Low
Tang et al., 2025 [27]	Cross-sectional	Participants who willingly took part in checkups at the Health Promotion Center of Sir Run Run Shaw Hospital Affiliated with Zhejiang University from 2020–2024	Renal ultrasonography for all participants during health screenings	142,309/10,605	42.4	57.2%	3.9%	13.0%	23.8	Categorical Continuous DRMA	Low
Qin et al., 2021 [28]	Cross-sectional	NHANES 2007–2014 (US population)	Self-reported history via the NHANES interview questionnaire (prevalent stones and recurrence from two survey questions).	20,972/1950	47.42	48.9%	9.1%	32.0%	28.8	Categorical Continuous	Low
Pan et al., 2023 [42]	Cross-sectional	Patients at Xinhua Hospital including 206 stone cases and 208 non-stone controls	Diagnosed clearly by imaging and surgery	414/206	52.55	50.5%	11.1%	23.4%	24.0	Categorical Continuous	Low
Lee et al., 2021 [41]	Cross-sectional	Participants in the Taiwan Biobank from 2008–2020	Main Taiwan Biobank cohort: self-reported history via interviewer-administered questionnaire (“Have you ever had kidney stones?”)	121,605/7738	49.88	35.9%	5.2%	12.2%	24.2	Categorical Continuous	Low
An et al., 2025 [35]	Prospective cohort	Adults aged 37–73 years from 22 assessment centers across England, Scotland, and Wales between 2006–2010 (UK Biobank)	The primary outcome of the study was the incidence of KSD. The ICD-10, OPCS-4, and 133 self-reported operation codes were used to record KSD diagnoses.	413,209/4551	56.53	46.1%	6.6%	55.6%	27.4	Categorical Continuous DRMA	Low
Cui et al., 2025 [40]	Cross-sectional	NHANES 2017–2020 (US population)	Self-reported doctor diagnosis via NHANES interview (prevalent stones): answered “yes” to “Has a doctor ever said you have kidney stones?”	2211/218	NA	49.8%	NA	57.9%	NA	Continuous	Low
Zhang et al., 2025 [30]	Cross-sectional	Adult patients who participated in physical examinations at the Health Management Center of Northern Jiangsu People’s Hospital from 2021–2023	Abdominal/renal ultrasound (Color Doppler) performed by certified sonographers; groups defined by ultrasound diagnosis; diagnostic criterion: echogenic foci/clusters in the kidney with acoustic shadowing.	43,891/2422	46.97	56.7%	6.6%	15.8%	24.2	Continuous	Low
Zhou et al., 2025 [45]	Cross-sectional	Adults aged 20–80 years who underwent health examinations at the Ruijin Hospital Health Examination Center 2020–2024	Abdominal/renal ultrasound (Color Doppler) performed by certified sonographers; groups defined by ultrasound diagnosis; diagnostic criterion: echogenic foci/clusters in kidney with acoustic shadowing.	18,868/991	44.99	61.4%	6.2%	27.8%	NA	Categorical Continuous DRMA	Low
Yang et al., 2025 [31]	Cross-sectional	NHANES 2007–2018 (US population)	Self-reported doctor diagnosis via responses to the question, ‘Have you ever had kidney stones?’	9605/829	45.7	48.8%	0%	33.9%	NA	DRMA	Low
Yuquan et al., 2025 [46]	Cross-sectional	Adults with type 2 diabetes mellitus hospitalized in the 900th hospital of the joint logistics support force from 2022–2023	Computed tomography shows high-density foci in the renal collecting system; ultrasound shows hyperechoic foci in the renal sinus with acoustic shadow; and X-ray shows round or staghorn dense opacities.	517/84	57.92	67.1%	100%	49.1%	24.6	Categorical Continuous DRMA	Low

Liu et al. (2026) [47], Pan et al. (2023) [42], An et al. (2025) [35], and Zhang et al. (2025) [30] were preprints/non-peer-reviewed reports at the time of their inclusion

*Abbreviations*: *DM* Diabetes mellitus, *HTN* Hypertension, *BMI* Body mass index, *ROB* Risk of bias, *DRMA* Dose-response meta-analysis, *NHANES* National Health and Nutrition Examination Survey, *KSD* Kidney stone disease, *ICD-10* International Classification of Diseases, 10th Revision, *OPCS-4* Office of Population Censuses and Surveys Classification of Interventions and Procedures, 4th Revision, *UK* United Kingdom, *US* United States

### TyG index (continuous) and kidney stone

**Fig. 2:**
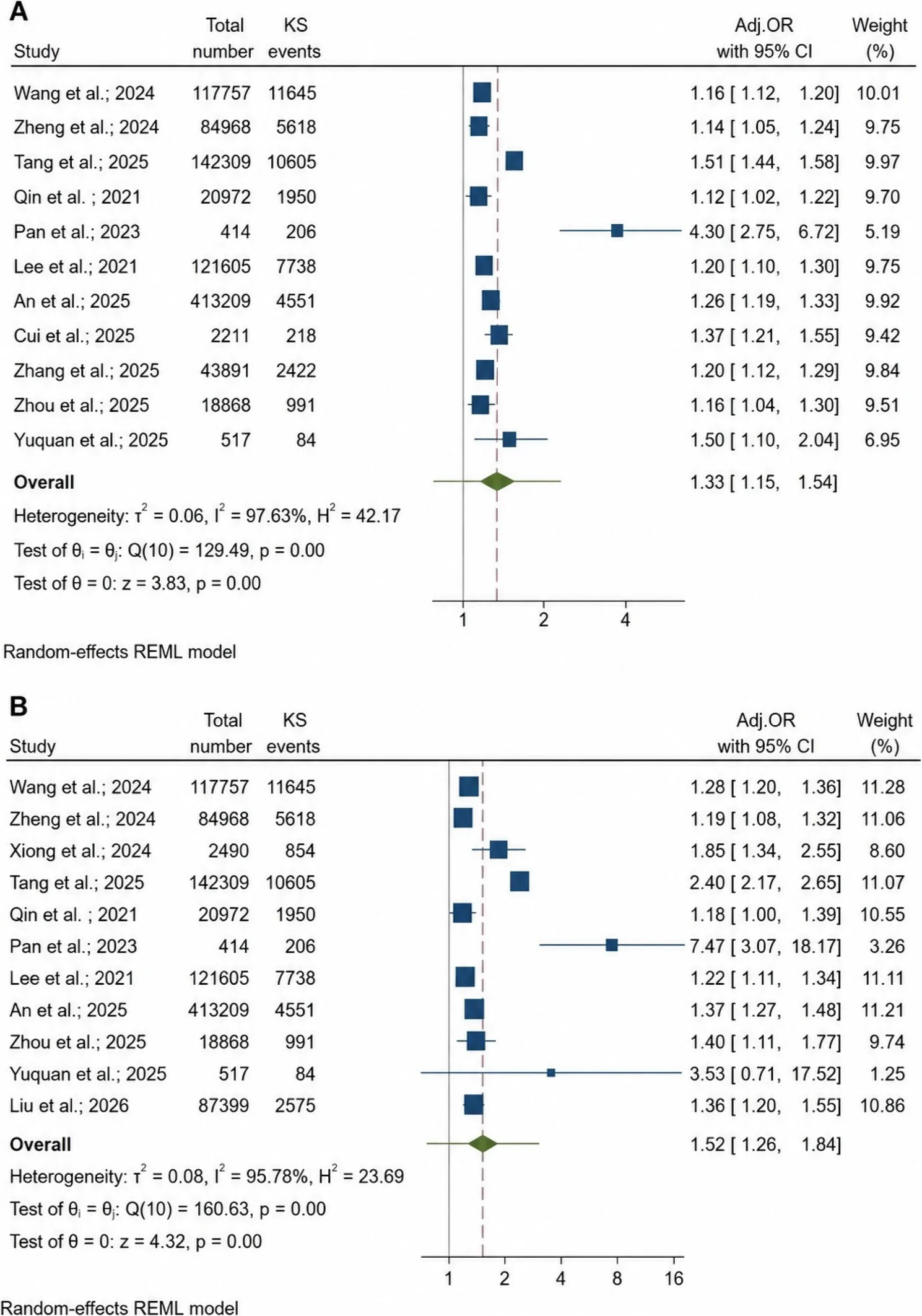
Association between the triglyceride-glucose (TyG) index and kidney stone disease (KSD) in (**A**) adjusted continuous analysis (**B**) adjusted categorical analysis. Note: The pooled diamond represents the overall random-effects adjusted odds ratio with its 95% confidence interval. The green horizontal line above the pooled diamond represents a 95% prediction interval. The overall pooled estimate was based on the total study weight (100%)

In the primary analysis of 11 studies reporting adjusted continuous associations, a 1-unit increase in TyG was associated with higher odds of KSD (pooled OR = 1.33; 95% CI: 1.15, 1.54) (Fig. 2A). Between-study heterogeneity was very high (I² = 97.6%), and the 95% prediction interval spanned 0.76 to 2.33.

The pre-specified subgroup analyses did not show any statistically significant between-group differences (Table 2). Subgroup analyses by region, diagnostic method, and study size yielded broadly consistent associations, with pooled estimates ranging from 1.23 to 1.58 and no evidence of effect modification (all tests for subgroup differences, *P* > 0.05). In a sensitivity analysis restricted to cross-sectional studies, the association remained (pooled OR: 1.39; 95% CI: 1.13, 1.71), although heterogeneity persisted. Similarly, restricting the analysis to studies reporting per 1-unit TyG effects (excluding per SD conversions) yielded a comparable pooled estimate. Sensitivity analyses excluding preprints/non-peer-reviewed reports showed that the results were materially unchanged (adjusted OR: 1.24; 95% CI: 1.14,1.35; I² = 89.8%). Leave-one-out analyses indicated that no single study materially influenced the findings, and the pooled OR was significant (Figure S1). The pooled association remained significant across all restriction-based sensitivity analyses (pooled adjusted ORs ranging from 1.24 to 1.79). Heterogeneity remained very high in all the restricted analyses (Table S7).

**Table 2:** Subgroup and sensitivity analyses for the association between TyG index (continuous) and kidney stones

Subgroup	Number of studies	Meta-analysis	Heterogeneity		Subgroup-difference test	
		Pooled adjusted OR (95% CI)	I^2^ within subgroup (%)	Between-group I² (%)	Qb	*P* between group
All	11	1.33 (1.15, 1.54)	97.63%			
Study population region				0%	0.83	0.36
East Asian populations	8	1.41 (1.10, 1.80)	98.89%			
Outside East Asia	3	1.24 (1.11, 1.38)	78.33%			
Total sample size				6.5%	1.07	0.30
<25,000	5	1.58 (1.10, 2.46)	97.86%			
>40,000	6	1.24 (1.14, 1.35)	92.59%			
Diagnostic method				13.8%	1.16	0.28
Imaging-based	7	1.45 (1.08, 1.96)	99.13%			
Self-report/ICD codes	4	1.23 (1.14, 1.32)	66.88%			
Study design						
Restricted to cross-sectional	9	1.39 (1.13, 1.71)	97.81%			
Restricted to per 1-unit reporting	8	1.43 (1.12, 1.83)	98.26%			
Excluding preprint studies	8	1.24 (1.14, 1.35)	89.81%			

I² within subgroup = residual between-study heterogeneity within each subgroup; between-group I² = heterogeneity attributable to subgroup differences derived from Qb; Qb = Q statistic for subgroup differences

*Abbreviations:* *TyG*  Triglyceride-glucose index, *OR*  Odds ratio, *CI*  Confidence interval, *I*² Between-study heterogeneity, *ICD*  International Classification of Diseases

Meta-regression showed no association between the effect size and mean age, percentage of men, or hypertension prevalence (all *P* > 0.05), and explained 0% of the heterogeneity (Table S9). In contrast, the crude synthesis was larger in magnitude (OR = 1.65, 95% CI 1.38, 1.98; I² = 99.3%) (Figure S2). Small-study effects were suggested by Egger’s test (*P* < 0.001), whereas Begg’s test was not significant (*P* = 0.35). A trim-and-fill check left the pooled estimates unchanged (Figure S3).

### High TyG index category and kidney stone

A comparison between the highest and lowest TyG index categories (k = 11) showed a pooled adjusted OR of 1.52 (95% CI: 1.26, 1.84) (Fig. 2B). Heterogeneity was very severe (I² = 95.8%), and the 95% prediction interval ranged from 0.77 to 3.02. Subgroups generally pointed in the same direction. There was no clear evidence of subgroup differences by region or study size, while the diagnostic-method comparison was borderline: East-Asia 1.64 (95% CI: 1.26, 2.13) versus outside East-Asia 1.29 (95% CI: 1.12, 1.49) (between-group *P* = 0.12), imaging-based diagnosis 1.74 (95% CI: 1.28, 2.37) versus self-report/ICD 1.27 (95% CI: 1.16, 1.40) (*P* = 0.05), and studies with < 25,000 participants 2.07 (95% CI: 1.11, 3.87) versus ≥ 40,000 participants 1.42 (95% CI: 1.15, 1.75) (*P* = 0.26). Limiting the analysis to cross-sectional studies produced a similarly elevated and significant estimate, and excluding preprints/non-peer-reviewed reports showed that higher TyG categories were still associated with increased odds of KSD (adjusted OR: 1.46, 95% CI: 1.19, 1.79; I² = 94.9%) (Table 3).

**Table 3:** Subgroup and sensitivity analyses for the association between TyG index (categorical) and kidney stones

Subgroup	Number of studies	Meta-analysis	Heterogeneity		Subgroup-difference test	
		Pooled adjusted OR (95% CI)	I^2^ within subgroup (%)	Between-group I² (%)	Qb	*P* between group
All	11	1.52 (1.26, 1.84)	95.78%			
Study population region				59.0%	2.44	0.12
East Asian populations	9	1.64 (1.26, 2.13)	96.87%			
Outside East Asia	2	1.29 (1.12, 1.49)	62.88%			
Total sample size				20.6%	1.26	0.26
<25,000	5	2.07 (1.11, 3.87)	93.86%			
>40,000	6	1.42 (1.15, 1.75)	97.01%			
Diagnostic method				73.0%	3.71	0.05
Imaging-based	8	1.74 (1.28, 2.37)	97.04%			
Self-report/ICD codes	3	1.27 (1.16, 1.40)	58.94%			
Study design						
Restricted to cross-sectional	9	1.63 (1.24, 2.14)	95.97%			
Excluding preprint studies	8	1.46 (1.19, 1.79)	94.90%			

I² within subgroup = residual between-study heterogeneity within each subgroup; between-group I² = heterogeneity attributable to subgroup differences derived from Qb; Qb = Q statistic for subgroup differences

*Abbreviations:* *TyG*  Triglyceride-glucose index, *OR*  Odds ratio, *CI*  Confidence interval, *I*² Between-study heterogeneity, *ICD*  International Classification of Diseases, *P*  p-value

The leave-one-out tests showed that no single study changed the direction or magnitude of the results (Figure S4). The association with KSD also remained statistically significant across all restriction-based sensitivity analyses (pooled adjusted ORs ranging from 1.45 to 2.08). Heterogeneity persisted at a very high level in each restricted analysis (Table S8). Meta-regression found no association between effect size and mean age, male proportion, or hypertension prevalence (all *P* > 0.05) (Table S9). Using crude estimates, the summary effect was higher (OR = 2.54, 95% CI 1.69, 3.80, I² = 99.28%) (Figure S5). The evidence for small-study effects was mixed. Egger’s test was significant (*P* = 0.0038), whereas Begg’s test did not show small-study effects (*P* = 0.0617). Trim-and-fill contour analysis left the pooled estimate unchanged, offering no evidence that small-study effects accounted for the association (Figure S6).

### Dose-response meta-analysis

**Fig. 3:**
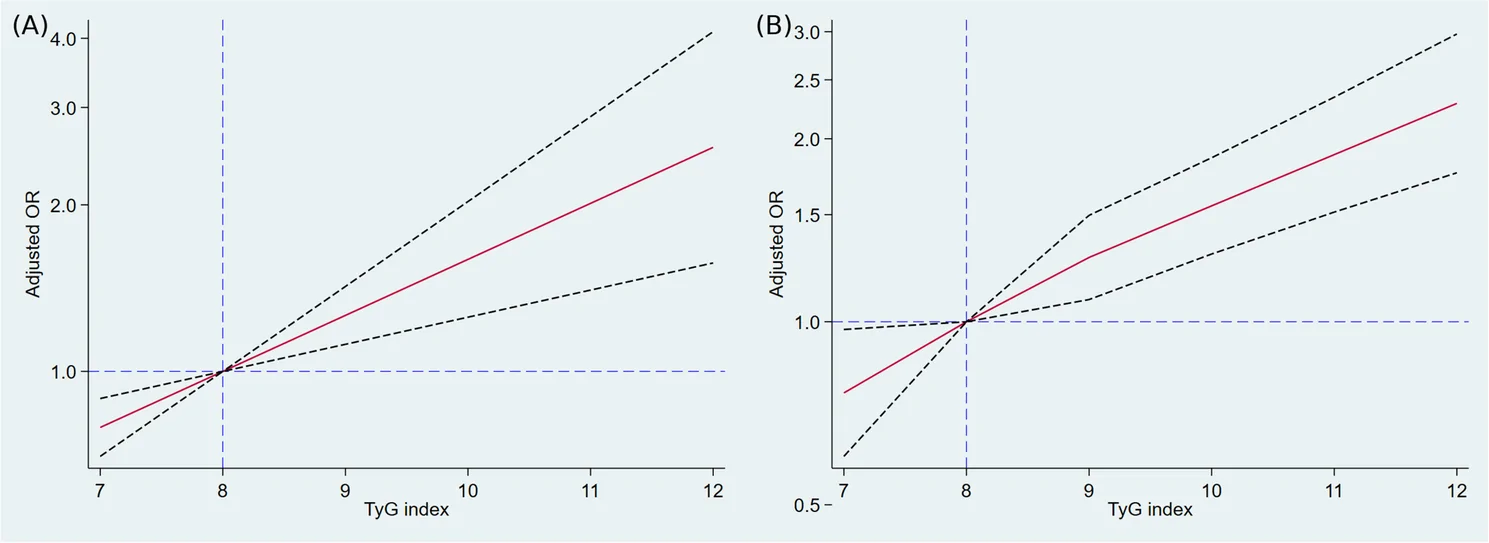
Dose-response relationship between the triglyceride-glucose (TyG) index and risk of kidney stones from a one-stage random-effects meta-analysis: (**A**) linear model and (**B**) non-linear trend assessed with a restricted cubic spline (three knots). The solid red line shows the pooled adjusted association (odds ratio, OR), and the black dashed lines show the 95% CIs. The blue horizontal dashed line indicates null (OR = 1.0). The vertical blue dashed line indicates the reference TyG value (= 8)

In a one-stage random-effects dose-response meta-analysis, eight studies contributing 30 TyG exposure categories were included, and higher TyG was positively associated with KSD [13, 27, 31, 35, 43–46]. The representative TyG values in the reference category ranged from 7.83 to 8.53, with a mean of approximately 8.01 (Table S10). In a one-stage random-effects dose-response meta-analysis, the association was compatible with a linear gradient, with a 26% increase in odds per 1-unit increase in TyG (OR: 1.26; 95% CI: 1.12, 1.42; 95% PI: 0.91, 1.75; *P* < 0.001, AIC = 3.22) (Fig. 3A). Although the RCS model with three knots provided a better overall fit than the linear model according to the AIC (AIC = -15.14 vs. 3.22; ΔAIC = 18.36), the Wald test showed no statistically significant evidence of non-linearity (*P* = 0.550) (Fig. 3B). Therefore, the association was interpreted as essentially linear, while acknowledging that the improved spline fit may reflect modest absorption of between-study variations rather than true curvature. The curves were centered at TyG = 8, consistent with the pre-specified calibration described in the Methods section. As a sensitivity check, the two-stage RCS dose-response model produced concordant estimates and did not alter the inference. In a sensitivity analysis excluding preprints, seven peer-reviewed studies remained in the linear one-stage dose-response meta-analysis. The association remained materially unchanged, with each 1-unit higher TyG still associated with increased odds of KSD (OR: 1.26; 95% CI: 1.09, 1.45).

On crude data, the one-stage random-effects model showed a steeper slope: each 1-unit increase in TyG was associated with greater KSD odds (OR = 1.68; 95% CI: 1.31, 2.16; *P* < 0.001) (Figure S7). A three-knot RCS produced a similar pattern with nonsignificant non-linearity (*P* = 0.074) and fit the data better than the linear model (AIC 3.64 vs. 210.01) (Figure S8). Both curves were centered at TyG = 8 and increased thereafter.

### Certainty of evidence (GRADE)

Using GRADE, the certainty of all analyses remained very low. Judgments were downgraded for substantial heterogeneity and possible small-study effects. The dose-response gradient was considered a potential upgrading domain; however, it did not result in a net increase in the final certainty rating (Table S11).

## Discussion

In this dose-response meta-analysis of 14 observational studies including 1,066,215 adults, a higher TyG was associated with higher odds of KSD. In the pooled adjusted analyses, each 1-unit increase in TyG corresponded to a 33% increase in the odds of KSD, and adults in the highest TyG category had 52% higher odds than those in the lowest category. In the one-stage dose-response meta-analysis, although the RCS model fit the data better by AIC, the absence of a significant non-linear component suggests that the overall association is better interpreted as approximately linear rather than clearly curvilinear, supporting a theoretical linear gradient with a 26% increase in odds per 1-unit rise in TyG. The steeper slopes observed in crude models, which attenuated after multivariable adjustment, suggest that part of the gradient is explained by shared cardiometabolic risk factors, while a residual graded association remains. Nonetheless, most data came from East Asian health-exam cohorts, which likely shaped the baseline risk and TyG distributions and contributed to the wide prediction intervals [27, 30, 41–45]. Taken together, the present findings synthesize the available observational evidence and support TyG as an IR-related marker that is positively associated with a higher risk of KSD.

These findings align with the results from large Chinese health examination cohorts, wherein higher TyG and several related IR indices, such as the atherogenic index of plasma (AIP), TyG-BMI, metabolic score for IR (METS-IR), and others, have been linked to higher kidney stone prevalence [27, 30, 42–45]. NHANES-based analyses in the United States have likewise found that TyG and TyG-derived indices are associated with higher KSD prevalence in nationally representative samples [28, 40], and an analysis from the UK Biobank documented similar qualitative patterns in a European population with prospective follow-up [35]. Complementing these findings, Zhou et al. showed that IR surrogates, including TyG, TyG-BMI, and METS-IR, mediated approximately 7–13% of the association between accelerated biological aging and KSD in a Chinese health examination cohort, supporting a shared metabolic aging pathway for stone formation [45].

In contrast, Yang et al., who restricted their NHANES sample to non-diabetic adults and fitted extensively adjusted models, observed an attenuation of the TyG index-stone association after full adjustment, underscoring the influence of the underlying IR range and covariate adjustment rather than contradicting the overall direction of the effect [31]. Studies conducted in clinical cohorts enriched for diabetes or metabolic derangements tended to report stronger associations than community-based samples [40, 46], consistent with a broader exposure and higher baseline IR burden. Overall, TyG and other IR indices appear to mark a “metabolic stone” phenotype, with stronger associations in metabolically high-risk groups and smaller, more adjustment-sensitive effects in lower-risk non-diabetic populations.

Several biologically plausible pathways may explain the association between higher TyG and KSD. Mechanistic and clinical studies suggest that IR may impair renal ammoniagenesis and distal acid excretion, lower urinary pH, and favor uric acid supersaturation and crystallization [17, 19, 48–50]. IR and the broader metabolic syndrome milieu have also been associated with more lithogenic urine profiles, including higher urinary calcium, uric acid, and oxalate, and lower citrate and effective urine volume [48, 50, 51]. Because TyG and related indices are validated surrogates of IR and cardiometabolic burden [22–26], these pathways provide biologically plausible explanations for the association observed here; however, they do not establish causality.

In addition, the “vascular hypothesis” of nephrolithiasis proposes that metabolic syndrome and IR may contribute to microvascular injury in the renal papillae, with subsequent repair leading to Randall’s plaque deposition in a process analogous to atherosclerotic plaque formation [52–55]. Emerging data also suggest that pro-inflammatory dietary patterns and altered gut-derived metabolites, such as short-chain fatty acids and tryptophan derivatives, may contribute to systemic inflammation and IR and are associated with more lithogenic urine profiles [42, 56, 57], although these pathways were not captured in the present meta-analysis. Finally, the near-linear dose-response that was observed is biologically compatible with a graded “metabolic stress” model; however, this pattern should be interpreted as biologically plausible and hypothesis-generating, not as evidence of causation.

### Clinical relevance and future perspectives

From a clinical perspective, these findings do not support the use of TyG as a stand-alone screening tool for KSD. Similar to the overall certainty rating, NHANES analyses have shown that TyG alone has only modest discrimination for prevalent KSD (area under the curve of approximately 0.57–0.58) [58, 59], which further argues against its use as a stand-alone screening test. At present, TyG is better viewed as a simple marker of adverse cardiometabolic status that may help risk-stratify patients in whom both metabolic optimization and standard stone-prevention measures warrant closer attention. In clinical care, an elevated TyG value may prompt a broader metabolic assessment and reinforce counseling on weight control, glycemic control, lipid management, hydration, diet, and other standard stone-prevention measures, particularly in patients with recurrent KSD or cardiometabolic comorbidities. Because the included studies were designed mainly to assess associations rather than to compare prediction models, this meta-analysis could not determine whether TyG provides incremental predictive value beyond established metabolic risk factors such as adiposity, glycemic status, dyslipidemia, and related renal risk markers. Nonetheless, the directionally consistent findings in the sensitivity analyses restricted to studies that adjusted for core covariates, glycemic measures, lipid measures, and even the TyG components themselves suggest that the observed association is not wholly explained by these factors, although this should not be interpreted as proof of the added predictive performance.

Future research should include prospective cohorts with repeated standardized TyG measurements, adjudicated incident KSD, and harmonized analytical plans. These studies should also collect stone composition and 24-h urine indices to test whether urine chemistry mediates the TyG-KSD association and explore prespecified effect modifications by sex, adiposity, diabetes status, and urine pH. TyG index should be evaluated alongside other IR surrogates in both etiological and prediction models, and contemporary causal inference approaches should clarify whether it acts mainly as a risk marker or a modifiable target. Ultimately, pragmatic and interventional studies are needed to determine whether a lower TyG index reduces the incidence or recurrence of KSD.

### Strengths and limitations

This review had several strengths. This is the first dose-response meta-analysis to synthesize the association between the TyG index and KSD. The analysis included a large sample size, used both continuous and categorical TyG estimates, and assessed the shape of the association through formal dose-response modelling. The robustness of the findings was also examined through extensive subgroup and sensitivity analyses, including detailed stratification by geographic region, diagnostic method, study size, study design, peer-review status, exposure scaling, and adjustment for key covariate domains, as well as meta-regression and small-study effect analyses.

Several overarching limitations should be considered when interpreting these findings. First, the evidence base is dominated by cross-sectional observational studies with relatively few incident outcomes, which constrain causal inference. Consequently, reverse causation cannot be excluded; for example, existing KSD or its correlated metabolic and behavioral consequences may influence TyG levels rather than TyG preceding stone development. Second, the very high between-study heterogeneity and very wide prediction intervals indicate that no single factor explains the dispersion. Instead, the heterogeneity likely reflects several clinical and methodological differences across studies, including variations in study populations and baseline metabolic risk, differences in TyG distributions and category cut-offs, heterogeneity in kidney stone ascertainment methods, inconsistent fasting status reporting and laboratory procedures, and substantial variations in covariate adjustment models. Some of these factors were formally explored through subgroup analyses, including diagnostic method, geographic region, and study size, as well as meta-regression and restriction-based sensitivity analyses. However, these approaches do not fully explain between-study variability. In categorical analyses, “highest” versus “lowest” TyG was also defined inconsistently across studies (for example, tertiles, quartiles, quintiles, or study-specific cut-offs), which further reduced the comparability. Third, residual confounding remains possible, particularly for metabolic comorbidities, adiposity, diet, and related cardiometabolic factors that were inconsistently measured or adjusted for across studies. Meta-regression to evaluate diabetes as an effect modifier could not be performed because too few studies reported diabetes-stratified effect estimates to support a reliable analysis, falling below the prespecified minimum number of studies (k < 5). Fourth, outcome misclassification is possible in self-reported and claims-based datasets, and both stone composition and urinary metabolic parameters (such as urine pH, citrate, calcium, oxalate, uric acid, and urine volume) were rarely reported, precluding phenotype-specific analyses and limiting mechanistic insight into how IR might relate to specific stone phenotypes or lithogenic urinary abnormalities. Finally, most participants were drawn from East Asian health-examination cohorts; therefore, generalizability to other populations is uncertain.

## Conclusion

The available evidence suggests that a higher TyG index is associated with greater KSD prevalence across continuous, categorical, and dose-response analyses, with an approximately linear gradient that is consistent with an IR-related metabolic pattern. Given the observational design, substantial heterogeneity, and differences in populations and outcome definitions, these findings should primarily refine hypotheses and guide prospective studies rather than justify immediate TyG-based utilization in clinical practice. Clinically, TyG may be most useful as a simple cardiometabolic risk marker that reinforces broader metabolic assessment, lifestyle counseling, and standard stone-prevention strategies in patients with KSD or metabolic risk factors. Future studies should prioritize prospective cohorts with adjudicated incident KSD, standardized stone phenotyping and urine chemistry, and explicit tests of effect modification. Ultimately, the practical value of TyG for KSD prevention depends on whether it adds meaningful predictive information beyond established risk factors and whether improvements in TyG are accompanied by a demonstrably lower KSD incidence or recurrence.

## Supplementary Information

Supplementary Material 1.

## Data Availability

All summarized data utilized in this meta-analysis can be found in the referenced publications and accompanying Supplementary Material.

## References

[1] Shastri S, Patel J, Sambandam KK, Lederer ED. Kidney Stone Pathophysiology, Evaluation and Management: Core Curriculum 2023. Am J Kidney Dis. 2023;82(5):617–34. 10.1053/j.ajkd.2023.03.017. 37565942 10.1053/j.ajkd.2023.03.017 PMC11370273

[2] Singh P, Harris PC, Sas DJ, Lieske JC. The genetics of kidney stone disease and nephrocalcinosis. Nat Rev Nephrol. 2022;18(4):224–40. 10.1038/s41581-021-00513-4. 34907378 10.1038/s41581-021-00513-4

[3] Khan SR, Pearle MS, Robertson WG, Gambaro G, Canales BK, Doizi S, et al. Kidney stones. Nat Rev Dis Primers. 2016;2:16008. 10.1038/nrdp.2016.8. 27188687 10.1038/nrdp.2016.8 PMC5685519

[4] Li Y, Di X, Liu M, Wei J, Li T, Liao B. Association between daily sitting time and kidney stones based on the National Health and Nutrition Examination Survey (NHANES) 2007–2016: a cross-sectional study. Int J Surg. 2024;110(8):4624–32. 10.1097/js9.0000000000001560. 38768465 10.1097/JS9.0000000000001560 PMC11325893

[5] Peerapen P, Thongboonkerd V. Kidney Stone Prevention. Adv Nutr. 2023;14(3):555–69. 10.1016/j.advnut.2023.03.002. 36906146 10.1016/j.advnut.2023.03.002 PMC10201681

[6] Rule AD, Krambeck AE, Lieske JC. Chronic kidney disease in kidney stone formers. Clin J Am Soc Nephrol. 2011;6(8):2069–75. 10.2215/cjn.10651110. 21784825 10.2215/CJN.10651110 PMC3156433

[7] Tang X, Bergstralh EJ, Mehta RA, Vrtiska TJ, Milliner DS, Lieske JC. Nephrocalcinosis is a risk factor for kidney failure in primary hyperoxaluria. Kidney Int. 2015;87(3):623–31. 10.1038/ki.2014.298. 25229337 10.1038/ki.2014.298 PMC4344931

[8] Tunnicliffe DJ, Wickham I, Jauré A, Johnston B, Mallett AJ, Mullan A, et al. Identifying and integrating consumer-prioritised topics and outcomes in clinical practice guidelines on managing kidney stones. BMC nephrology. 2025;26(1):292. 10.1186/s12882-025-04160-w PMC12211754 40597765

[9] Hyams ES, Matlaga BR. Economic impact of urinary stones. Transl Androl Urol. 2014;3(3):278–83. 10.3978/j.issn.2223-4683.2014.07.02. 26816777 10.3978/j.issn.2223-4683.2014.07.02 PMC4708578

[10] Rule AD, Lieske JC, Pais VM. Jr. Management of Kidney Stones in 2020. JAMA. 2020;323(19):1961–2. 10.1001/jama.2020.0662. 32191284 10.1001/jama.2020.0662

[11] Carbone A, Al Salhi Y, Tasca A, Palleschi G, Fuschi A, De Nunzio C, et al. Obesity and kidney stone disease: a systematic review. Minerva Urol Nefrol. 2018;70(4):393–400. 10.23736/s0393-2249.18.03113-2. 29856171 10.23736/S0393-2249.18.03113-2

[12] Wong Y, Cook P, Roderick P, Somani BK. Metabolic Syndrome and Kidney Stone Disease: A Systematic Review of Literature. J Endourol. 2016;30(3):246–53. 10.1089/end.2015.0567. 26576717 10.1089/end.2015.0567

[13] Wang D, Zhang D, Zhang L, Shi F, Zhu Y. Association between triglyceride-glucose index and risk of kidney stone: a Chinese population-based case–control study. BMJ open. 2024;14(11):e086641. 39578031 10.1136/bmjopen-2024-086641 PMC11590796

[14] Wang DW, Shi F, Zhang DG, Wang H, Zhu Y, Wang J. Remnant cholesterol increases the risk of incident kidney stones: a nested case-control study in Chinese adults. Urolithiasis. 2024;52(1):160. 10.1007/s00240-024-01658-0. 39540945 10.1007/s00240-024-01658-0

[15] Wang D, Shi F, Zhang D, Wang H, Zhu Y, Wang J. The atherogenic index of plasma increases the risk of incident kidney stones: a nested case-control study in Chinese adults. Ren Fail. 2025;47(1):2458757. 10.1080/0886022x.2025.2458757. 39904806 10.1080/0886022X.2025.2458757 PMC11800335

[16] Tarplin S, Ganesan V, Monga M. Stone formation and management after bariatric surgery. Nat Rev Urol. 2015;12(5):263–70. 10.1038/nrurol.2015.67. 25850790 10.1038/nrurol.2015.67

[17] Spatola L, Ferraro PM, Gambaro G, Badalamenti S, Dauriz M. Metabolic syndrome and uric acid nephrolithiasis: insulin resistance in focus. Metabolism. 2018;83:225–33. 10.1016/j.metabol.2018.02.008. 29510180 10.1016/j.metabol.2018.02.008

[18] Kim S, Chang Y, Jung HS, Hyun YY, Lee KB, Joo KJ, et al. Glycemic Status, Insulin Resistance, and the Risk of Nephrolithiasis: A Cohort Study. Am J Kidney Dis. 2020;76(5):658–e681. 10.1053/j.ajkd.2020.03.013. 32534797 10.1053/j.ajkd.2020.03.013

[19] Spatola L, Angelini C, Badalamenti S, Maringhini S, Gambaro G. Kidney stones diseases and glycaemic statuses: focus on the latest clinical evidences. Urolithiasis. 2017;45(5):457–60. 10.1007/s00240-016-0956-8. 27921141 10.1007/s00240-016-0956-8

[20] Rahman IA, Nusaly IF, Syahrir S, Nusaly H, Mansyur MA. Association between metabolic syndrome components and the risk of developing nephrolithiasis: A systematic review and bayesian meta-analysis. F1000Res. 2021;10:104. 10.12688/f1000research.28346.1. 34804491 10.12688/f1000research.28346.1 PMC8577060

[21] Besiroglu H, Otunctemur A, Ozbek E. The metabolic syndrome and urolithiasis: a systematic review and meta-analysis. Ren Fail. 2015;37(1):1–6. 10.3109/0886022X.2014.976133. 25353628 10.3109/0886022X.2014.976133

[22] Khan SH, Sobia F, Niazi NK, Manzoor SM, Fazal N, Ahmad F. Metabolic clustering of risk factors: evaluation of Triglyceride-glucose index (TyG index) for evaluation of insulin resistance. Diabetol Metab Syndr. 2018;10(1):74. 10.1186/s13098-018-0376-8. 30323862 10.1186/s13098-018-0376-8 PMC6173832

[23] Guerrero-Romero F, Simental-Mendía LE, González-Ortiz M, Martínez-Abundis E, Ramos-Zavala MG, Hernández-González SO, et al. The product of triglycerides and glucose, a simple measure of insulin sensitivity. Comparison with the euglycemic-hyperinsulinemic clamp. J Clin Endocrinol Metab. 2010;95(7):3347–51. 10.1210/jc.2010-0288. 20484475 10.1210/jc.2010-0288

[24] Simental-Mendía LE, Rodríguez-Morán M, Guerrero-Romero F. The product of fasting glucose and triglycerides as surrogate for identifying insulin resistance in apparently healthy subjects. Metab Syndr Relat Disord. 2008;6(4):299–304. 10.1089/met.2008.0034. 19067533 10.1089/met.2008.0034

[25] Lopez-Jaramillo P, Gomez-Arbelaez D, Martinez-Bello D, Abat MEM, Alhabib KF, Avezum Á, et al. Association of the triglyceride glucose index as a measure of insulin resistance with mortality and cardiovascular disease in populations from five continents (PURE study): a prospective cohort study. Lancet Healthy Longev. 2023;4(1):e23–33. 10.1016/s2666-7568(22)00247-1. 36521498 10.1016/S2666-7568(22)00247-1

[26] Chen N, Ma LL, Zhang Y, Chu X, Dong J, Yan YX. Association of long-term triglyceride-glucose index patterns with the incidence of chronic kidney disease among non-diabetic population: evidence from a functional community cohort. Cardiovasc Diabetol. 2024;23(1):7. 10.1186/s12933-023-02098-7. 38172903 10.1186/s12933-023-02098-7 PMC10765660

[27] Tang Y, Cai X, Chen L. Exploring the relationship between triglyceride-glucose index and kidney stones in Chinese adults: a cross-sectional study. Translational Androl Urol. 2025;14(6):1542. 10.21037/tau-2025-143 PMC12271952 40687643

[28] Qin Z, Zhao J, Geng J, Chang K, Liao R, Su B. Higher triglyceride–glucose index is associated with increased likelihood of kidney stones. Front Endocrinol. 2021;12:774567. 10.3389/fendo.2021.774567 PMC8667164 34912299

[29] Jiang H, Li L, Liu J, Xu B, Chen S, Zhu W, et al. Triglyceride-Glucose Index as a Novel Biomarker in the Occurrence of Kidney Stones: A Cross-Sectional Population-Based Study. Int J Gen Med. 2021;14:6233–44. 10.2147/ijgm.S334821. 34616176 10.2147/IJGM.S334821 PMC8487863

[30] Zhang S, Liang H, Liu J, Zhu Y. The Association Between the ZJU Index, Metabolic Dysfunction-Associated Steatotic Liver Disease, and Nephrolithiasis in Adult Men: A Cross-sectional Study. 2025. 10.21203/rs.3.rs-6867840/v1.

[31] Yang Y-X, Xiang J-C, Ye G-C, Luo K-D, Wang S-G, Xia Q-D. Association of insulin resistance indices with kidney stones and their recurrence in a non-diabetic population: an analysis based on NHANES data from 2007–2018. Ren Fail. 2025;47(1):2490203. 40275575 10.1080/0886022X.2025.2490203 PMC12035944

[32] Page MJ, McKenzie JE, Bossuyt PM, Boutron I, Hoffmann TC, Mulrow CD, et al. The PRISMA 2020 statement: an updated guideline for reporting systematic reviews. BMJ. 2021;372:n71. 10.1136/bmj.n71. 33782057 10.1136/bmj.n71 PMC8005924

[33] Moola S, Munn Z, Tufanaru C, Aromataris E, Sears K, Sfetcu R, et al. Systematic reviews of etiology and risk. JBI Man Evid synthesis. 2020;1:217–69.

[34] Saletta J, Garcia J, Caramês JMM, Schliephake H, da Silva Marques D. Quality assessment of systematic reviews on vertical bone regeneration. Int J Oral Maxillofac Surg. 2019;48(3):364–72. 30139710 10.1016/j.ijom.2018.07.014

[35] An R, Cui Z-y, Feng L, Song L, Song Z, Song B et al. Triglyceride-Glucose Index, Genetic Susceptibility, and the Risk of Kidney Stone Disease: A Prospective Cohort Study. Available at 10.2139/ssrn.5242651. SSRN 5242651.

[36] Orsini N, Li R, Wolk A, Khudyakov P, Spiegelman D. Meta-analysis for linear and nonlinear dose-response relations: examples, an evaluation of approximations, and software. Am J Epidemiol. 2012;175(1):66–73. 22135359 10.1093/aje/kwr265 PMC3244608

[37] Harrell FE. Introduction. In: Harrell JFE, editor. Regression Modeling Strategies: With Applications to Linear Models, Logistic and Ordinal Regression, and Survival Analysis. Cham: Springer International Publishing; 2015. pp. 1–11.

[38] Guyatt G, Oxman AD, Akl EA, Kunz R, Vist G, Brozek J, et al. GRADE guidelines: 1. Introduction—GRADE evidence profiles and summary of findings tables. J Clin Epidemiol. 2011;64(4):383–94. 21195583 10.1016/j.jclinepi.2010.04.026

[39] Murad MH, Verbeek J, Schwingshackl L, Filippini T, Vinceti M, Akl EA, et al. GRADE guidance 38: updated guidance for rating up certainty of evidence due to a dose-response gradient. J Clin Epidemiol. 2023;164:45–53. 37777140 10.1016/j.jclinepi.2023.09.011

[40] Cui Y, Wu A, Liu H, Zhong Y, Yi K. The effect and potential mechanisms of per-and polyfluoroalkyl substances (PFAS) exposure on kidney stone risk. Ecotoxicol Environ Saf. 2025;294:118087. 40157329 10.1016/j.ecoenv.2025.118087

[41] Lee M-R, Ke H-L, Huang J-C, Huang S-P, Geng J-H. Obesity-related indices and its association with kidney stone disease: a cross-sectional and longitudinal cohort study. Urolithiasis. 2022;50(1):55–63. 34714367 10.1007/s00240-021-01288-w

[42] Pan Y, Xu Y, Zhang L, Huang Y, Qian S, Xu G. Clinical Lipid Metabolism Correlates with Urinary Calculus: TyG Index Has a Closer Correlation. Research Square. 2023. 10.21203/rs.3.rs-2904148/v1.

[43] Xiong ZJ, He YM, Yuan P, He FK, Yu KF, Liu B. Study on the correlation between triglyceride glucose product index and kidney stones. Mod Prev Med. 2024;51(21):4022–6. 10.20043/j.cnki.MPM.202406136.

[44] Zheng S, Hua T, Yin G, Zhang W, Yao Y, Li Y. Association between the triglyceride-glucose index and the incidence of nephrolithiasis in male individuals. Beijing da xue xue bao Yi xue ban=. J Peking Univ Health Sci. 2024;56(4):610–6. 10.19723/j.issn.1671-167X.2024.04.011 PMC11284483 39041554

[45] Zhou Z, Gong Z, Lu G, Huang B, Wang D. Association between biological aging acceleration and kidney stone in Chinese adults: exploring the role of insulin resistance. Urolithiasis. 2025;53(1):178. 40965671 10.1007/s00240-025-01857-3

[46] Chen YQ, Zeng H, Lin L, Chen P, Yi Y, Xu B. 2型糖尿病患者甘油三酯-葡萄糖指数与肾结石的相关性研究 [Correlation between triglyceride-glucose index and kidney stones in patients with type 2 diabetes mellitus]. Chinese Journal of Diabetes Mellitus. 2025;17(9):1166–1175. 10.3760/cma.j.cn115791-20250125-00039.

[47] Liu Y, Zhang J, Zheng X. Association of triglyceride-glucose index and its combined obesity indicators and kidney stones in the non-diabetic population of southwestern China. 2026. 10.21203/rs.3.rs-8643580/v1.

[48] Li H, Klett DE, Littleton R, Elder JS, Sammon JD. Role of insulin resistance in uric acid nephrolithiasis. World J Nephrol. 2014;3(4):237–42. 10.5527/wjn.v3.i4.237. 25374817 10.5527/wjn.v3.i4.237 PMC4220356

[49] Ramaswamy K, Shah O. Metabolic syndrome and nephrolithiasis. Translational Androl Urol. 2014;3(3):285–95. 10.3978/j.issn.2223-4683.2014.06.03 PMC4708567 26816779

[50] Hess B. Metabolic syndrome, obesity and kidney stones. Arab J Urol. 2012;10(3):258–64. 10.1016/j.aju.2012.04.005. 26558034 10.1016/j.aju.2012.04.005 PMC4442970

[51] Kohjimoto Y, Sasaki Y, Iguchi M, Matsumura N, Inagaki T, Hara I. Association of Metabolic Syndrome Traits and Severity of Kidney Stones: Results From a Nationwide Survey on Urolithiasis in Japan. Am J Kidney Dis. 2013;61(6):923–9. 10.1053/j.ajkd.2012.12.028. 23433467 10.1053/j.ajkd.2012.12.028

[52] Taylor ER, Stoller ML. Vascular theory of the formation of Randall plaques. Urolithiasis. 2015;43(Suppl 1):41–5. 25475492 10.1007/s00240-014-0718-4

[53] Stoller ML, Meng MV, Abrahams HM, Kane JP. The primary stone event: a new hypothesis involving a vascular etiology. J Urol. 2004;171(5):1920–4. 15076312 10.1097/01.ju.0000120291.90839.49

[54] Khan SR, Canales BK, Dominguez-Gutierrez PR. Randall’s plaque and calcium oxalate stone formation: role for immunity and inflammation. Nat Rev Nephrol. 2021;17(6):417–33. 33514941 10.1038/s41581-020-00392-1

[55] Bagga HS, Chi T, Miller J, Stoller ML. New insights into the pathogenesis of renal calculi. Urologic Clin. 2013;40(1):1–12. 10.1016/j.ucl.2012.09.006 PMC4165395 23177630

[56] Maddahi N, Yarizadeh H, Aghamir SMK, Alizadeh S, Yekaninejad MS, Mirzaei K. The association of dietary inflammatory index with urinary risk factors of kidney stones formation in men with nephrolithiasis. BMC Res Notes. 2020;13(1):373. 32771046 10.1186/s13104-020-05206-y PMC7414556

[57] Prezioso D, Strazzullo P, Lotti T, Bianchi G, Borghi L, Caione P, et al. Dietary treatment of urinary risk factors for renal stone formation. A review of CLU Working Group. Archivio Italiano di Urol Andrologia. 2015;87(2):105–20. 10.4081/aiua.2015.2.105 26150027

[58] Yu H, Wu J. Associations of triglyceride glucose-related parameters with kidney stones: a cross-sectional study from NHANES 2007–2020. Translational Androl Urol. 2025;14(2):379. 10.21037/tau-24-516 PMC11921200 40114842

[59] Liu M, Yang P, Gou Y. Association between triglyceride glucose index-related indices and kidney stones in adults based on NHANES 2007–2020. Front Endocrinol. 2025;15:1516982. 10.3389/fendo.2024.1516982 PMC11746126 39839481

