# Supplementary material for: Association between the triglyceride-glucose index and kidney stone disease: a systematic review and dose-response meta-analysis
Source: Lipids Health Dis. 2026 Jun 12;25:183. doi: 10.1186/s12944-026-02994-3 (PMC13495508; doi:10.1186/s12944-026-02994-3)
Supplement: Supplementary file 1 [file 12944_2026_2994_MOESM1_ESM.docx]

**Table of Contents**

[Table S1. Search strategy	2](#_Toc225693772)

[Table S2. Summary of fasting-status reporting across the included studies	4](#_Toc225693773)

[Table S3. Results of risk of bias assessment for cross-sectional studies using JBI checklist for analytical cross-sectional studies.	5](#_Toc225693774)

[Table S4. Results of risk of bias assessment for cohort studies using JBI checklist for analytical cohort studies.	6](#_Toc225693775)

[Table S5. Results of risk of bias assessment for case-control studies using JBI checklist for analytical case-control studies.	7](#_Toc225693776)

[Table S6. Covariates included in the maximally adjusted model used for adjusted meta-analysis (per study).	8](#_Toc225693777)

[Abbreviations:	8](#_Toc225693778)

[Table S7. Sensitivity analyses for the association between TyG index (continuous) and kidney stone across different covariate adjustment sets.	9](#_Toc225693779)

[Table S8. Sensitivity analyses for the association between categorical TyG index and kidney stone across different covariate adjustment sets.	10](#_Toc225693780)

[Table S9. Random-effects meta-regression analyses of continuous and categorical TyG associations with kidney stone disease.	11](#_Toc225693781)

[Table S10. Study-level data contributing to the dose-response meta-analysis	12](#_Toc225693782)

[Table S11. GRADE Summary of Findings for the association between TyG index and kidney stones.	13](#_Toc225693783)

[Figure S1. Leave-one-out influence analysis for continuous TyG (per 1-unit) and kidney stone risk.	14](#_Toc225693784)

[Figure S2. Forest plot of crude odds ratios for the association between continuous TyG (per 1-unit) and kidney stone risk.	15](#_Toc225693785)

[Figure S3. Funnel plot for small-study effects in the meta-analysis of adjusted odds ratios (continuous TyG, per 1-unit).	16](#_Toc225693786)

[Figure S4. Leave-one-out influence analysis for high TyG category (highest vs lowest) and kidney stone risk.	17](#_Toc225693787)

[Figure S5. Forest plot of crude odds ratios for the association between high TyG category (highest vs lowest) and kidney stone risk.	18](#_Toc225693788)

[Figure S6. Funnel plot for small-study effects in the meta-analysis of adjusted odds ratios (high TyG category, highest vs lowest).	19](#_Toc225693789)

[Figure S7. Dose-response between the triglyceride–glucose (TyG) index and risk of kidney stones under a *linear* one-stage random-effects model (crude estimates).	20](#_Toc225693790)

[Figure S8. Dose-response between the TyG index and risk of kidney stones under a *nonlinear* restricted cubic spline model with three knots (crude estimates).	21](#_Toc225693791)

## **Table S1. Search strategy**

| **Databases** | **Date** | **Field** | **Search query** |
| --- | --- | --- | --- |
| PubMed | 05/22/2026 | All fields | ("Kidney Calculi"[Mesh] OR "Urolithiasis"[Mesh] OR "Nephrolithiasis"[Mesh] OR "Nephrocalcinosis"[Mesh] OR "nephrolithiasis" OR "urolithiasis" OR "nephrocalcinosis" OR "renal lithiasis" OR "kidney stone*" OR "renal stone*" OR "kidney calculi" OR "renal calculi" OR "kidney calculus" OR "renal calculus" OR "urinary calculi" OR "urinary tract stone*") AND ("Triglyceride-Glucose index" OR "Triglyceride Glucose index" OR "glucose-triglyceride index" OR "Triglyceride and glucose index" OR "glucose and triglycerides index" OR "Triglyceride/glucose index" OR "TyG index" OR "TyG" OR "TyGI" OR "triacylglycerol glucose index" OR "Triglyceride-Glucose related indices" OR "TYG indices" OR "insulin resistance index" OR "insulin resistance indices" OR "insulin resistance marker*" OR "insulin resistance surrogate*") |
| EMBASE | 05/22/2026 | All fields | ('nephrolithiasis'/exp OR 'urolithiasis'/exp OR 'nephrocalcinosis'/exp OR 'renal lithiasis' OR 'kidney stone*' OR 'renal stone*' OR 'kidney calculi' OR 'renal calculi' OR 'kidney calculus' OR 'renal calculus' OR 'urinary calculi' OR 'urinary tract stone*') AND ('triglyceride glucose index' OR 'triglyceride-glucose index' OR 'glucose-triglyceride index' OR 'triglyceride and glucose index' OR 'glucose and triglycerides index' OR 'triglyceride/glucose index' OR 'tyg index' OR 'tyg' OR 'tygi' OR 'triacylglycerol glucose index' OR 'triglyceride-glucose related indices' OR 'tyg indices' OR 'insulin resistance index' OR 'insulin resistance indices' OR 'insulin resistance marker*' OR 'insulin resistance surrogate*') |
| Web of Science | 05/22/2026 | All fields | (ALL=(("nephrolithiasis" OR "urolithiasis" OR "nephrocalcinosis" OR "renal lithiasis" OR "kidney stone*" OR "renal stone*" OR "kidney calculi" OR "renal calculi" OR "kidney calculus" OR "renal calculus" OR "urinary calculi" OR "urinary tract stone*"))) AND ALL=( ("Triglyceride Glucose index" OR "Triglyceride-Glucose index" OR "glucose-triglyceride index" OR "Triglyceride and glucose index" OR "glucose and triglycerides index" OR "Triglyceride/glucose index" OR "TyG index" OR "TyG" OR "TyGI" OR "glucose and triglycerides" OR "Triglyceride-Glucose" OR "triacylglycerol glucose index" OR "triacylglycerol glucose index" OR "Triglyceride-Glucose related indices" OR "TYG indices" OR "insulin resistance index" OR "insulin resistance indices" OR "insulin resistance marker*" OR "insulin resistance surrogate*")) |
| Scopus | 05/22/2026 | All fields | ALL ( ( "nephrolithiasis" OR "urolithiasis" OR "nephrocalcinosis" OR "renal lithiasis" OR "kidney stone*" OR "renal stone*" OR "kidney calculi" OR "renal calculi" OR "kidney calculus" OR "renal calculus" OR "urinary calculi" OR "urinary tract stone*" ) AND ( "Triglyceride Glucose index" OR "Triglyceride-Glucose index" OR "glucose-triglyceride index" OR "Triglyceride and glucose index" OR "glucose and triglycerides index" OR "Triglyceride/glucose index" OR "TyG index" OR "TyG" OR "TyGI" OR "glucose and triglycerides" OR "Triglyceride-Glucose" OR "triacylglycerol glucose index" OR "triacylglycerol glucose index" OR "Triglyceride-Glucose related indices" OR "TYG indices" OR "insulin resistance index" OR "insulin resistance indices" OR "insulin resistance marker*" OR "insulin resistance surrogate*" ) ) |
| Cochrane Reviews | 05/22/2026 | All fields | (nephrolithiasis OR urolithiasis OR nephrocalcinosis OR "renal lithiasis" OR (kidney NEXT stone*) OR (renal NEXT stone*) OR "kidney calculi" OR "renal calculi" OR "kidney calculus" OR "renal calculus" OR "urinary calculi" OR (urinary NEXT tract NEXT stone*)) AND ("insulin resistance" OR "metabolic syndrome") |
| ProQuest | 05/22/2026 | All fields | ("nephrolithiasis" OR "urolithiasis" OR "nephrocalcinosis" OR "renal lithiasis" OR "kidney stone*" OR "renal stone*" OR "kidney calculi" OR "renal calculi" OR "kidney calculus" OR "renal calculus" OR "urinary calculi" OR "urinary tract stone*") AND ("Triglyceride-Glucose index" OR "Triglyceride Glucose index" OR "glucose-triglyceride index" OR "Triglyceride and glucose index" OR "glucose and triglycerides index" OR "Triglyceride/glucose index" OR "TyG index" OR "TyG" OR "TyGI" OR "triacylglycerol glucose index" OR "Triglyceride-Glucose related indices" OR "TYG indices" OR "insulin resistance index" OR "insulin resistance indices" OR "insulin resistance marker*" OR "insulin resistance surrogate*") |
| Manual search in Google and Google Scholar | 05/22/2026 |  | combinations of the main keywords (e.g. “kidney stones”, “urolithiasis”, “nephrolithiasis”, “triglyceride-glucose index”, “TyG index”, “insulin resistance surrogate”). We screened at least the first 200 Google Scholar records and the first 9-10 Google result pages for potentially eligible observational studies, conference papers, theses, and Chinese/grey literature not indexed in the major databases. |

## Table S2. Summary of fasting-status reporting across the included studies

| **Study** | **Fasting-status reporting** |
| --- | --- |
| **Liu et al., 2026** | Protocol not explicitly reported / unclear |
| **Wang et al., 2024** | Explicitly verified (overnight fasting blood samples collected) |
| **Tang et al., 2025** | Explicitly verified (fasting venous blood; fasting defined as >8 h since last meal) |
| **Qin et al., 2021** | Fasting biomarkers used, but protocol not explicitly reported |
| **Yang et al., 2025** | Fasting subsample/weights used (NHANES WTSAF2YR restriction and fasting subsample weights) |
| **Yuquan et al., 2025** | Fasting biomarkers used, but protocol not explicitly reported |
| **An et al., 2025** | Fasting biomarkers used, but protocol not explicitly reported |
| **Pan et al., 2023** | Protocol not explicitly reported / unclear |
| **Zhang et al., 2025** | Protocol not explicitly reported / unclear |
| **Zheng et al., 2024** | Protocol not explicitly reported / unclear |
| **Zhou et al., 2025** | Protocol not explicitly reported / unclear |
| **Xiong et al., 2024** | Protocol not explicitly reported / unclear |
| **Cui et al., 2025** | Protocol not explicitly reported / unclear |
| **Lee et al., 2021** | Protocol not explicitly reported / unclear |

## Table S3. Results of risk of bias assessment for cross-sectional studies using JBI checklist for analytical cross-sectional studies.

| **First Author, year** | **Item number** | | | | | | | | **Total score** | **Risk of Bias** |
| --- | --- | --- | --- | --- | --- | --- | --- | --- | --- | --- |
|  | **1** | **2** | **3** | **4** | **5** | **6** | **7** | **8** |  |  |
| Liu et al. (2026) | Yes | Yes | Yes | Yes | Yes | Yes | Yes | Yes | 8 | Low |
| Xiong et al. (2024) | Yes | Yes | Yes | Yes | Yes | Yes | Yes | Yes | 8 | Low |
| Tang et al. (2025) | Yes | Yes | Yes | Yes | Yes | Yes | Yes | Yes | 8 | Low |
| Qin et al. (2021) | Yes | Yes | Yes | No | Yes | Yes | Unclear | Yes | 6 | Low |
| Pan et al. (2023) | Yes | Yes | Unclear | Yes | Yes | Yes | Yes | Yes | 7 | Low |
| Cui et al. (2025) | Yes | Yes | Yes | No | Yes | Yes | Unclear | yes | 6 | Low |
| Zhou et al. (2025) | Yes | Yes | Yes | Yes | Yes | Yes | Yes | Yes | 8 | Low |
| Zheng et al. (2024) | Yes | Yes | Yes | Yes | Yes | Yes | Yes | Yes | 8 | Low |
| Zhang et al. (2025) | Yes | Yes | Yes | Yes | Yes | Yes | Yes | Yes | 8 | Low |
| Lee et al. (2021) | Yes | Yes | Yes | No | Yes | Yes | Unclear | Yes | 6 | Low |
| Yuquan et al. (2025) | Yes | Yes | Yes | No | Yes | Yes | Yes | Yes | 8 | Low |
| Yang et al. (2025) | Yes | Yes | Yes | Yes | Yes | Yes | Unclear | Yes | 7 | Low |

1. Clear inclusion criteria of the sample
2. Sufficiency of description of the study subjects and settings
3. Validity and reliability of the measurement of exposure
4. The presence of objective criteria for measurement of the condition
5. Identification of confounding factors
6. Strategies to deal with the confounding factors
7. Validity and reliability of the outcome measurement
8. Appropriateness of the statistical analysis

## Table S4. Results of risk of bias assessment for cohort studies using JBI checklist for analytical cohort studies.

| **First Author, year** | **Item number** | | | | | | | | | | | **Total score** | **Risk of Bias** |
| --- | --- | --- | --- | --- | --- | --- | --- | --- | --- | --- | --- | --- | --- |
|  | **1** | **2** | **3** | **4** | **5** | **6** | **7** | **8** | **9** | **10** | **11** |  |  |
| An et al. (2025) | Yes | Yes | Yes | Yes | Yes | Yes | Yes | Yes | Unclear | No | Yes | 9 | Low |

1. Similarity between the two groups
2. Appropriateness of the measurement of the exposure to assign people to groups
3. Validity and reliability of the measurement of the exposure
4. Identification of confounding factors
5. Strategies to deal with the confounding factors
6. Absence of the outcomes of interest at the start of the study
7. Validity and reliability of the outcome measurement
8. Appropriateness of the duration of the follow-up
9. Completeness of the follow-up
10. Strategies to address incomplete follow-up
11. Appropriateness of the statistical analysis

## Table S5. Results of risk of bias assessment for case-control studies using JBI checklist for analytical case-control studies.

| **First Author, year** | **Item number** | | | | | | | | | | **Total score** | **Risk of Bias** |
| --- | --- | --- | --- | --- | --- | --- | --- | --- | --- | --- | --- | --- |
|  | **1** | **2** | **3** | **4** | **5** | **6** | **7** | **8** | **9** | **10** |  |  |
| Wang et al. (2024) | Yes | Yes | Yes | Yes | Yes | Yes | Yes | Yes | Unclear | Yes | 9 | Low |

1. Similarity between cases and controls
2. Appropriateness of matching between cases and controls
3. Consistent criteria for identifying cases and controls
4. Valid and reliable exposure measurement
5. Same exposure measurement for cases and controls
6. Identification of confounding factors
7. Strategies to control confounding
8. Valid and reliable outcome ascertainment
9. Adequacy of the exposure window
10. Appropriateness of the statistical analysis

## **Table S6.** Covariates included in the maximally adjusted model used for adjusted meta-analysis (per study).

| **First Author** | **Analysis** | **Covariates included in the model (as reported)** |
| --- | --- | --- |
| Liu et al., 2026 | Adjusted categorical analysis | Age, sex, smoking, drinking, HTN, calcium, HbA1c, eGFR, serum uric acid |
| Wang et al., 2024 | Adjusted continuous analysis  Adjusted categorical analysis | Age, sex and BMI, hypertension, diabetes, LDLC, serum uric acid and eGFR |
| Zheng et al., 2024 | Adjusted continuous analysis  Adjusted categorical analysis | Age, BMI category, hypertension, Scr, TC, HDL-c, LDL-c, and serum uric acid |
| Xiong et al., 2024 | Adjusted categorical analysis | Age, sex, uric acid, TC, SBP, DBP, HDL-C, LDL-C |
| Tang et al., 2025 | Adjusted continuous analysis  Adjusted categorical analysis | Age, gender, hypertension, diabetes, smoking, drinking, BMI, SBP, DBP, HbA1c, HDL, TC, LDL, serum uric acid, CRP, and BUN. |
| Qin et al., 2021 | Adjusted continuous analysis  Adjusted categorical analysis | Gender, age, race, education level, poverty-to-income ratio, marital status, alcohol intake, physical activity, cholesterol, BMI, smoking status, hypertension, and diabetes. |
| Pan et al., 2023 | Adjusted continuous analysis  Adjusted categorical analysis | Age, sex, BMI, glucose, diabetic and HTN incidence |
| Lee et al., 2021 | Adjusted continuous analysis  Adjusted categorical analysis | Age, sex, smoking status, alcohol status, exercise habits, SBP, DBP, history of hypertension, diabetes, and dyslipidemia, eGFR, uric acid, Hemoglobin, albumin, FBS, HbA1c and TG |
| An et al., 2025 | Adjusted continuous analysis  Adjusted categorical analysis | Age, sex, ethnicity, education, Townsend deprivation index, physical activity, smoking status, drinking status, water intake, diet score, e-GFR, diabetes and hypertension. |
| Cui et al., 2025 | Adjusted continuous analysis | Age, sex, ethnicity, poverty-to-income ratio, BMI, smoking status, drinking status, physical levels, hypertension, and cardiovascular disease. |
| Zhang et al., 2025 | Adjusted continuous analysis | hypertension, diabetes, MASLD, WBC, PLT, ALB, RBC, Scr, ALT, and AST, age, BMI, and smoking |
| Zhou et al., 2025 | Adjusted continuous analysis  Adjusted categorical analysis | Age, sex, hypertension, diabetes, and hyperuricemia. |
| Yuquan et al., 2025 | Adjusted continuous analysis  Adjusted categorical analysis | BUN, Scr, serum uric acid, FBS, TC, TG, HDL-c, LDL-c, serum calcium ions, urine albumin/creatinine ratio, HbA1c, fasting C-peptide, urine pH, fasting insulin, and duration of diabetes. |

Abbreviations: HTN = hypertension; BMI = body mass index; LDL-C = low-density lipoprotein cholesterol; HDL-C = high-density lipoprotein cholesterol; TC = total cholesterol; TG = triglycerides; SBP = systolic blood pressure; DBP = diastolic blood pressure; Scr = serum creatinine; eGFR = estimated glomerular filtration rate; HbA1c = glycated hemoglobin; CRP = C-reactive protein; BUN = blood urea nitrogen; FBS = fasting blood sugar; MASLD = metabolic dysfunction-associated steatotic liver disease; WBC = white blood cell count; PLT = platelet count; ALB = albumin; RBC = red blood cell count; ALT = alanine aminotransferase; AST = aspartate aminotransferase; UACR = urine albumin/creatinine ratio; IR = insulin resistance.

## Table S7. Sensitivity analyses for the association between TyG index (continuous) and kidney stone across different covariate adjustment sets.

| **Sensitivity analysis (restriction based on adjustment set)** | **Number of studies** | **Pooled adjusted OR (95%CI)** | **I^2^ (%)** |
| --- | --- | --- | --- |
| **All (full-adjusted)** | 11 | 1.33 (1.15, 1.54) | 97.63% |
| **Restricted to studies adjusting for core^¶^ covariates (age, sex, BMI)** | 5 [13, 27, 28, 40, 42] | 1.58 (1.02, 2.44) | 99.50% |
| **Restricted to studies that adjust for kidney function markers^†^** | 7 [13, 27, 30, 39, 41, 44, 46] | 1.25 (1.15, 1.36) | 91.25% |
| **Restricted to studies that adjust for glycemic measures^‡^** | 4 [27, 41, 42, 46] | 1.79 (1.06, 3.03) | 98.82% |
| **Restricted to studies that adjust for uric acid/hyperuricemia^€^** | 6 [13, 27, 41, 44-46] | 1.25 (1.13, 1.38) | 92.02% |
| **Restricted to studies that adjust for lipid panel^Ω^** | 7 [13, 27, 28, 41, 42, 44, 46] | 1.24 (1.12, 1.38) | 92.90% |
| **Restricted to studies that did not adjust for TyG components (FBS and TG)** | 9 [13, 27, 28, 30, 39, 40, 42, 44, 45] | 1.35 (1.11, 1.66) | 98.70% |

^¶^ **Core covariates:** age, sex, and BMI.

**† Kidney function markers:** eGFR, serum creatinine, BUN, or UACR.

**^‡^ Glycemic measures:** fasting glucose/FBS, HbA1c, insulin (± C-peptide).

**^€^ Uric acid:** serum uric acid and/or hyperuricemia.

**^Ω^ Lipid panel:** TC, HDL, LDL, or TG.

**Abbreviations:** BMI = body mass index; eGFR = estimated glomerular filtration rate; BUN = blood urea nitrogen; UACR = urinary albumin-to-creatinine ratio; FBS = fasting blood sugar; HbA1c = glycated hemoglobin; TC = total cholesterol; HDL = high-density lipoprotein cholesterol; LDL = low-density lipoprotein cholesterol; TG = triglycerides; TyG = triglyceride-glucose index; OR = odds ratio; CI = confidence interval.

## Table S8. Sensitivity analyses for the association between categorical TyG index and kidney stone across different covariate adjustment sets.

| **Sensitivity analysis (restriction based on adjustment set)** | **Number of studies** | **Pooled adjusted OR (95%CI)** | **I^2^ (%)** |
| --- | --- | --- | --- |
| **All (full-adjusted)** | 11 | 1.52 (1.26, 1.84) | 95.78% |
| **Restricted to studies adjusting for core^¶^ covariates (age, sex, BMI)** | 4 [13, 27, 28, 42] | 2.08 (1.01, 4.31) | 99.24% |
| **Restricted to studies that adjust for kidney function markers^†^** | 7 [13, 27, 41, 44-47] | 1.45 (1.17, 1.79) | 95.53% |
| **Restricted to studies that adjust for glycemic measures^‡^** | 5 [27, 41, 42, 45, 47] | 1.90 (1.13, 3.17) | 98.39% |
| **Restricted to studies that adjust for uric acid/hyperuricemia^€^** | 8 [13, 27, 39, 41, 43-45, 47] | 1.45 (1.23, 1.73) | 95.27% |
| **Restricted to studies that adjust for lipid panel^Ω^** | 7 [13, 27, 28, 41, 43-45] | 1.44 (1.17, 1.77) | 95.58% |
| **Restricted to studies that did not adjust for TyG components (FBS and TG)** | 9 [13, 27, 28, 39, 42-44, 46, 47] | 1.44 (1.21, 1.73) | 94.08% |

^¶^ **Core covariates:** age, sex, and BMI.

**† Kidney function markers:** eGFR, serum creatinine, BUN, or UACR.

**^‡^ Glycemic measures:** fasting glucose/FBS, HbA1c, insulin (± C-peptide).

**^€^ Uric acid:** serum uric acid and/or hyperuricemia.

**^Ω^ Lipid panel:** TC, HDL, LDL, or TG.

**Abbreviations:** BMI = body mass index; eGFR = estimated glomerular filtration rate; BUN = blood urea nitrogen; UACR = urinary albumin-to-creatinine ratio; FBS = fasting blood sugar; HbA1c = glycated hemoglobin; TC = total cholesterol; HDL = high-density lipoprotein cholesterol; LDL = low-density lipoprotein cholesterol; TG = triglycerides; TyG = triglyceride-glucose index; OR = odds ratio; CI = confidence interval.

## Table S9. Random-effects meta-regression analyses of continuous and categorical TyG associations with kidney stone disease.

| **Analysis** | **Moderator** | **k** | **Coefficient** | **95% CI** | **P value** | **R² (%)** |
| --- | --- | --- | --- | --- | --- | --- |
| **Continuous TyG** | Mean age | 10 | 1.011 | 0.972 to 1.052 | 0.577 | 0.00 |
|  | Male proportion (%) | 11 | 1.002 | 0.980 to 1.023 | 0.884 | 0.00 |
|  | Hypertension prevalence (%) | 11 | 0.999 | 0.988 to 1.010 | 0.863 | 0.00 |
| **Categorical TyG** | Mean age | 11 | 0.991 | 0.955 to 1.029 | 0.650 | 0.00 |
|  | Male proportion (%) | 10 | 1.008 | 0.994 to 1.022 | 0.272 | 8.90 |
|  | Hypertension prevalence (%) | 9 | 0.997 | 0.988 to 1.006 | 0.534 | 0.00 |

**Abbreviations:** CI, confidence interval; k, number of studies; R², proportion of between-study heterogeneity explained by the moderator.

***Note:*** *Meta-regression was performed using random-effects models with restricted maximum likelihood (REML).*

## **Table S10. Study-level data contributing to the dose-response meta-analysis**

| **First author, year** | **No. of TyG categories contributed** | **Reference category** | **Representative TyG value in reference category** | **Representative TyG values in higher categories** |
| --- | --- | --- | --- | --- |
| Wang et al., 2024 | 4 | Q1: <8.21 | 7.97 | 8.41, 8.79, 9.36 |
| Zheng et al., 2024 | 3 | T1: <8.34 | 8.08 | 8.61, 9.14 |
| Xiong et al., 2024 | 4 | Q1: 7.27–8.48 | 7.88 | 8.72, 9.23, 11.05 |
| An et al., 2025 | 3 | T1 | 8.12 | 8.68, 9.35 |
| Tang et al., 2025 | 4 | Q1 | 7.85 | 8.32, 8.73, 9.43 |
| Zhou et al., 2025 | 5 | Q1: <8.05 | 7.83 | 8.22, 8.51, 8.84, 9.36 |
| Yuquan et al., 2025 | 3 | Q1: <8.88 | 8.53 | 9.23, 9.93 |
| Yang et al., 2025 | 4 | Q1 | 7.83 | 8.32, 8.70, 9.31 |

Footnote: Eight studies contributed a total of 30 TyG exposure categories to the dose-response meta-analysis. The representative TyG value in the reference category ranged from 7.83 to 8.53, with a mean of approximately 8.01.

## **Table S11. GRADE Summary of Findings for the association between TyG index and kidney stones.**

| **Outcome** | **Studies (k)** | **OR, 95% CI)** | **Corresponding risk† (per 1,000)** | **Downgrades** | | | | | **Upgrades** | | | **Overall certainty** |
| --- | --- | --- | --- | --- | --- | --- | --- | --- | --- | --- | --- | --- |
|  |  |  |  | **Risk of bias** | **Inconsistency** | **Indirectness** | **Imprecision** | **Publication bias** | **Dose-response gradient** | **Large effects** | **Residual confounding** |  |
| **Kidney stones per 1-unit higher TyG** | 11 | 1.33 (1.15, 1.54) | 64 (56–74) | - | ↓ | - | - | ↓ | - | - | - | **Very low** (⊕◯◯◯) |
| **Kidney stones, highest vs lowest TyG** | 11 | 1.52 (1.26, 1.84) | 74 (61–92) | - | ↓ | - | - | ↓ | - | - | - | **Very low** (⊕◯◯◯) |

**† Crude prevalence of kidney stone based on the included studies was 4.7%**

**GRADE symbols:** ⊕⊕⊕⊕ High; ⊕⊕⊕◯ Moderate; ⊕⊕◯◯ Low; ⊕◯◯◯ Very low.

## Figure S1. Leave-one-out influence analysis for continuous TyG (per 1-unit) and kidney stone risk.

## Figure S2. Forest plot of crude odds ratios for the association between continuous TyG (per 1-unit) and kidney stone risk.

## Figure S3. Funnel plot for small-study effects in the meta-analysis of adjusted odds ratios (continuous TyG, per 1-unit).

Figure S4. Leave-one-out influence analysis for high TyG category (highest vs lowest) and kidney stone risk.

## Figure S5. Forest plot of crude odds ratios for the association between high TyG category (highest vs lowest) and kidney stone risk.

## Figure S6. Funnel plot for small-study effects in the meta-analysis of adjusted odds ratios (high TyG category, highest vs lowest).

## **Figure S7**. Dose-response between the triglyceride–glucose (TyG) index and risk of kidney stones under a *linear* one-stage random-effects model (crude estimates).

*The red line is the pooled OR; black dashed lines are 95% CIs. The blue horizontal dashed line marks OR = 1.0, and the vertical dashed line marks the reference TyG = 8.*

## **Figure S8**. Dose-response between the TyG index and risk of kidney stones under a *nonlinear* restricted cubic spline model with three knots (crude estimates).

*The red line is the pooled OR; black dashed lines are 95% CIs. The blue horizontal dashed line marks OR = 1.0, and the vertical dashed line marks the reference TyG = 8.*
